# In Vivo Magic Angle Magnetic Resonance Imaging for Cell Tracking in Equine Low-Field MRI

**DOI:** 10.1155/2019/5670106

**Published:** 2019-12-17

**Authors:** Carolin Horstmeier, Annette B. Ahrberg, Dagmar Berner, Janina Burk, Claudia Gittel, Aline Hillmann, Julia Offhaus, Walter Brehm

**Affiliations:** ^1^Department for Horses, Faculty of Veterinary Medicine, University of Leipzig, An den Tierkliniken 21, 04103 Leipzig, Germany; ^2^Department of Orthopedics, Traumatology, and Plastic Surgery, University of Leipzig, Liebigstr. 20, 04103 Leipzig, Germany; ^3^Royal Veterinary College, University of London, Hawkshead Lane, Hatfield, Hertfordshire AL9 7TA, UK; ^4^Equine Clinic-Surgery, Justus Liebig University Giessen, Frankfurter Str. 108, 35392 Giessen, Germany; ^5^Department of Veterinary Medicine, Queen's Veterinary School, Madingley Road, Cambridge CB3 0ES, UK; ^6^Saxon Incubator for Clinical Translation, University of Leipzig, Philipp-Rosenthal-Str. 55, 04103 Leipzig, Germany

## Abstract

The magic angle effect increases the MRI signal of healthy tendon tissue and could be used for more detailed evaluation of tendon structure. Furthermore, it could support the discrimination of hypointense artefacts induced by contrast agents such as superparamagnetic iron oxide used for cell tracking. However, magic angle MRI of the equine superficial digital flexor tendon has not been accomplished in vivo in standing low-field MRI so far. The aim of this in vivo study was to evaluate the practicability of this magic angle technique and its benefit for tracking superparamagnetic iron oxide-labelled multipotent mesenchymal stromal cells. Six horses with induced tendinopathy in their forelimb superficial digital flexor tendons were injected locally either with superparamagnetic iron oxide-labelled multipotent mesenchymal stromal cells or serum. MRI included standard and magic angle image series in T1- and T2∗-weighted sequences performed at regular intervals. Image analysis comprised blinded evaluation and quantitative assessment of signal-to-noise ratio. The magic angle technique enhanced the tendon signal-to-noise ratio (*P* < 0.001). Hypointense artefacts were observable in the cell-injected superficial digital flexor tendons over 24 weeks and artefact signal-to-noise ratio differed significantly from tendon signal-to-noise ratio in the magic angle images (*P* < 0.001). Magic angle imaging of the equine superficial digital flexor tendon is feasible in standing low-field MRI. The current data demonstrate that the technique improves discrimination of superparamagnetic iron oxide-induced artefacts from the surrounding tendon tissue.

## 1. Introduction

In equine practice, tendinopathies most commonly affect the superficial digital flexor tendon (SDFT) [1, 2] and treatment is challenging. Consequently, the SDFT is the focus of research activities aimed at understanding tendon healing and regenerative therapy options.

Standing low-field magnetic resonance imaging (MRI) offers good opportunities to closely monitor tendon healing, especially by using T2 and STIR sequences. It also allows longitudinal cell tracking after injecting superparamagnetic iron oxide- (SPIO-) labelled cells by the induction of hypointense artefacts based on inhomogeneities of the local magnetic field in T1- and T2∗-weighted sequences. Tendon signal is dependent on a particular angle *θ* between the tendon fibres and the main magnetic field *B*_0_ [3]. The angle *θ* = 90° in standard MRI images leads to a rapid dephasing of the MR signal, resulting in a hypointense signal of healthy tendon tissue. The so-called magic angle effect with an angle of approximately *θ* = 55° could be useful to improve the value of the diagnostic procedure. The T2 relaxation time is extended, and the tendon signal intensity (SI) increases [3, 4]. The magic angle effect is an artefact and can occur naturally in tendons and ligaments during MRI which could lead to misdiagnosis. But considering special clinical and scientific problems, it can provide additional information on the structure of tendons [5, 6] and could be advantageous for the discrimination of artefacts induced by contrast agents such as SPIO from the surrounding tissue [7, 8]. Intralesional injection of multipotent mesenchymal stromal cells (MSC) has been established with promising results [9–11]. However, little is known about the behaviour of the cells after injection. Therefore, MRI tracking would be favourable to monitor the fate of MSC and the healing process of the tendon lesions. However, while SPIO-labelled cells can be localised and distinguished in most tissues, a clear distinction between labelled cells and healthy tendon tissue is hampered as both display hypointense signals in standard images. Therefore, it appears favourable to use the magic angle technique to increase the tendon signal and support the discrimination of the labelled MSC after injection into the tendon. This beneficial effect of the magic angle has already been confirmed ex vivo [12, 13] and in vivo in rabbits [14], but it has not been performed in large animal in vivo studies so far [7, 8, 15]. Burk et al. in 2013 have shown that the magic angle effect is feasible in the SDFT of the midmetacarpal region.

We hypothesised that magic angle images can be obtained from the equine SDFT in standing low-field MRI. Furthermore, we aimed to evaluate the benefit of the thereby established technique for cell tracking in tendon lesions, hypothesising that the magic angle effect (*θ* = 55°) improves visualisation of labelled cells inside the tendon tissue compared to standard (*θ* = 90°) MRI.

## 2. Materials and Methods

### 2.1. Tendinopathy Induction

This study was approved by the local ethics committee (Landesdirektion Leipzig, TVV 34/13), and all ethical guidelines were observed. Six healthy standardbreds (3 female and 3 male, 3–10 years, 400–550 kg) were included.

Tendon lesions were induced by the same surgeon in the midmetacarpal region of the SDFT in both forelimbs under general anaesthesia. The horses were placed in lateral recumbency, and the surgical fields were prepared aseptically. Via a 2 cm vertical skin incision, an 11-gauge bone marrow aspiration needle (Walter Veterinär-Instrumente e.K., Baruth/Mark, Germany) was advanced 2 cm in the proximal direction into the SDFT. During retraction of the needle, 0.4 ml collagenase I (4.8 mg/ml; Life Technologies GmbH, Darmstadt, Germany) was injected into the tendon tissue. Subsequently, peritendineum and skin were sutured and the limbs were bandaged. Postoperative management included standardised pain management with nonsteroidal anti-inflammatory drugs (flunixin meglumine; pre- and 10 h postsurgery: 1.1 mg/kg bwt i.v.; day 1 to day 4 postsurgery: 0.55 mg/kg bwt p.o. twice daily; and day 5 to day 6 post surgery: 0.55 mg/kg bwt p.o. once daily). The horses were assessed for pain by a pain scoring system [16] 3 times daily over a period of 10 days after surgery and, if necessary, additional analgesics were administered.

### 2.2. MSC Isolation and Labelling

In the same surgery, subcutaneous adipose tissue was collected from the supragluteal region for the isolation of autologous MSC as described previously [17, 18]. The plastic-adherent cell fraction was expanded until passage 2 and was labelled with SPIO (Molday ION Rhodamine B™; iron concentration: 25 *μ*g Fe/ml culture medium, incubation: 20 h; BioPAL, Inc., Worcester, MA, USA). The MSC were harvested and 10 × 10^6^ cells per treated tendon were resuspended in 1 ml autologous serum for the intralesional injection. Labelled MSC from each animal were also used to confirm labelling by Prussian Blue staining and flow cytometry, trilineage differentiation potential, and MSC surface marker expression [19].

### 2.3. MSC Injection

Three weeks after induction of tendinopathy, treatment of the tendon lesions was performed. 10 × 10^6^ resuspended MSC were injected intralesionally into 1 randomly chosen forelimb of each animal. 1 ml autologous serum was injected as a control into the contralateral forelimb. For this purpose, horses were sedated and perineural anaesthesia of the ulnar nerve and local anaesthesia were performed. The skin was prepared aseptically. A 20-gauge needle was placed in the tendon lesion under ultrasonographic monitoring (10 MHz linear transducer, LOGIQ 5 Expert; GE Healthcare, Munich, Germany), and the cell suspension or serum was injected by the same blinded veterinarian. From week 2 to week 24 after cell injection, the horses were subjected to an increasing exercise program [20].

### 2.4. MRI

For MSC tracking over 24 weeks, the SDFT of both forelimbs were examined in standing, sedated animals using an equine-dedicated low-field MRI system (0.27 Tesla MRI unit; Hallmarq Veterinary Imaging, Guildford, Surrey, UK) as described before [8]. Examinations were performed immediately before and after cell injection as well as 1, 2, 3, 4, 6, 8, 12, and 24 weeks postinjection. Each examination included transversal T1-weighted and T2∗-weighted gradient echo MRI sequences (Table 1), both as a standard series as well as a magic angle series. In the standard series, the whole injured area was scanned. In the magic angle series of the MSC-treated forelimb, imaging was limited to the area with SPIO-induced hypointense artefacts, and in the magic angle series of the contralateral forelimb, 8 slices were acquired from the region of the injection.

**Figure 1:**
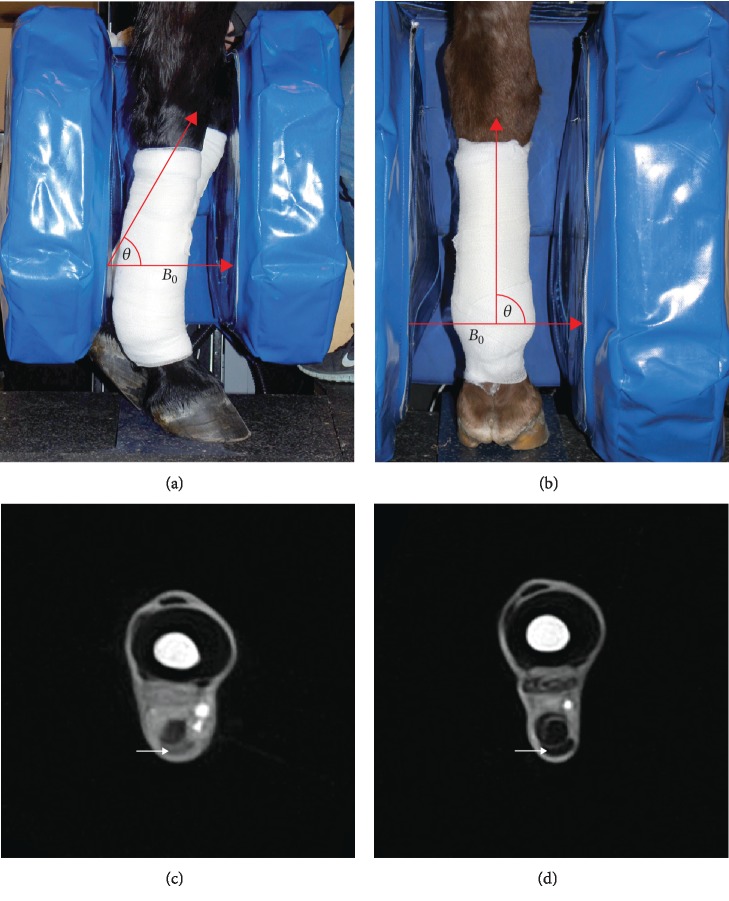
Position of the equine limb in the MRI scanner. (a) and (b) show the equine limb positioned between the poles of an equine-dedicated low-field MRI system. In (a), the SDFT of a left forelimb (posterior limb) was positioned in approximately the magic angle (*θ* = 55°) to the main magnetic field *B*_0_. In (b), the SDFT of a right forelimb was positioned in a *θ* = 90° angle (standard series) to the main magnetic field *B*_0_. (c) and (d) show T1-weighted magic angle (c) and standard (d) images of healthy SDFT (arrows) in the midmetacarpal region. The signal intensity of the SDFT in the magic angle image increases compared to the SDFT in the standard image.

For the standard series, the poles of the MRI framed the metacarpus on its medial and lateral side resulting in an angle of *θ* = 90° between the SDFT and the static magnetic field *B*_0_. For the magic angle series, the SDFT was positioned at an angle of approximately *θ* = 55° to the magnetic field *B*_0_. To accomplish this, the animal was moved sideways into the magnet, leaving the poles of the MRI dorsal and palmar to the metacarpal region, while the carpus was bent and fixed manually by one person (Figure 1).

### 2.5. Image Analysis

**Figure 2:**
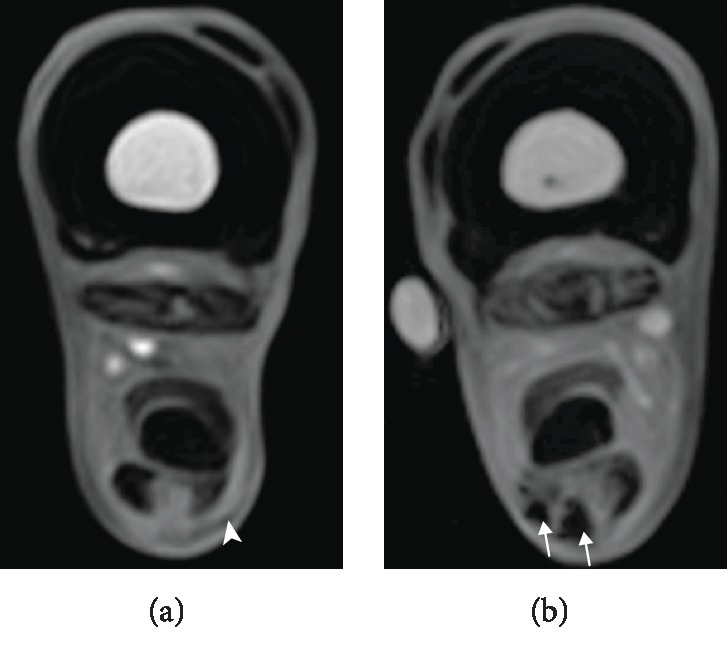
Categorisation of hypointense artefacts. Sample images of T1-weighted gradient echo MRI sequences with potential hypointense artefacts. (a) The arrowhead indicates a region of a category 2 artefact (“questionable hypointense area”). (b) The arrows indicate a region of a category 1 artefact (“hypointense area most likely related to SPIO”).

Image analysis was performed using Synedra View Personal Version 3.4.0.2 (Synedra Information Technologies GmbH, Innsbruck, Austria) and Mathematica 10.1 (Wolfram Research, Inc., Champaign, IL, USA). All T1- and T2∗-weighted standard and magic angle image series were randomized, and the presence of a potential hypointense artefact in each image was determined qualitatively in consensus by two blinded observers. First of all, images displaying potential artefacts were assigned to one of two categories (category 1: “hypointense area most likely related to SPIO”; category 2: “questionable hypointense area”) on the base of position, shape, and intensity (for example see Figure 2). Category 1 artefacts generally were characterised by lower SI and a larger area and were often localised in and around the SDFT. After this, only images with a potential hypointense artefact (categories 1 and 2) were used for further analysis. The SI of these artefacts were measured using a region of interest (circular ROI: 1 mm^2^). Additionally, the artefact area and its SI were measured based on the whole visible artefact area. In all images assigned to category 1, the surrounding SDFT SI were measured additionally. In standard images assigned to category 1, tendon lesion SI were determined using a region of interest (circular ROI: 1 mm^2^). This was not possible in magic angle images because of the difficult delineation between the tendon lesion and the surrounding tendon tissue. All measurements were repeated three times by a single observer, and mean values were used for further analysis. Furthermore, the standard deviation (SD) of the background noise, based on the whole background areas, was obtained.

For further analysis of all category 1 images, the signal-to-noise ratios of the different structures (SNR = SI/SD background) and the contrast-to-noise ratios (CNR) (CNR = (SI SDFT − SI artefact)/SD background) were used. In contrast, in category 2 images, only the artefact SNR were calculated for comparison. After all, the data were unblinded and the selected categorisation was evaluated. The artefact volumes displayed by all category 1 images within the whole limb series (*V* = *Σ* artefact areas × 6 mm; 6 mm representing a slice thickness of 5 mm and a gap of 1 mm) were calculated.

### 2.6. Histology

Within the framework of a different part of this study [19], histological sections of the tendons were obtained from the same horses at week 24 after euthanasia. Sections from the treated tendon were used to confirm that SPIO-labelled MSC were still present based on the rhodamine B component of the labelling agent. Briefly, the sections were subjected to counterstaining of nuclei with DAPI (Carl Roth GmbH & Co. KG, Karlsruhe, Germany). Other samples were stained with Prussian Blue with nuclear fast red counterstaining (Carl Roth GmbH & Co. KG, Karlsruhe, Germany) to evaluate the presence of fibroblast-like structures. All images were recorded using a Pannoramic Scan II (3DHISTECH Ltd., Budapest, Hungary). DAPI-stained sections were evaluated qualitatively by two observers in consensus, and Prussian Blue-stained sections were evaluated quantitatively by two blinded, independent observers.

### 2.7. Statistical Analysis

Statistical analysis was performed using SPSS® Statistics 22 (IBM, Ehningen, Germany). For comparisons of SI within the same image, the Wilcoxon test was used; for comparisons between different images, Mann-Whitney's *U* tests were performed. *P* values < 0.05 were considered significant.

## 3. Results

### 3.1. Magic Angle Effect

**Figure 3:**
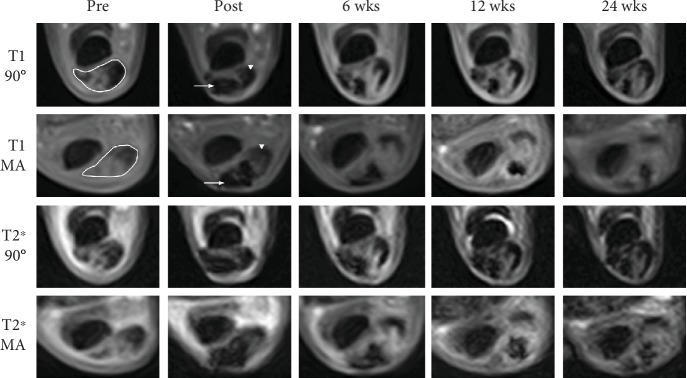
Exemplary MRI images. Exemplary images of T1- and T2∗-weighted gradient echo MRI sequences before and immediately after as well as 6, 12, and 24 weeks after cell application at the same level of the limb. The first and third rows show standard images (90°), and the second and fourth rows show the corresponding magic angle (MA) images. The SDFT with its hyperintense lesion is identified by the white edging. After injection of labelled cells, they appear as pronounced hypointense artefacts (white arrows). Note that the healthy tendon tissue (white arrowheads) is difficult to discriminate from the artefact in *θ* = 90° images but not in the magic angle (MA) images.

**Figure 4:**
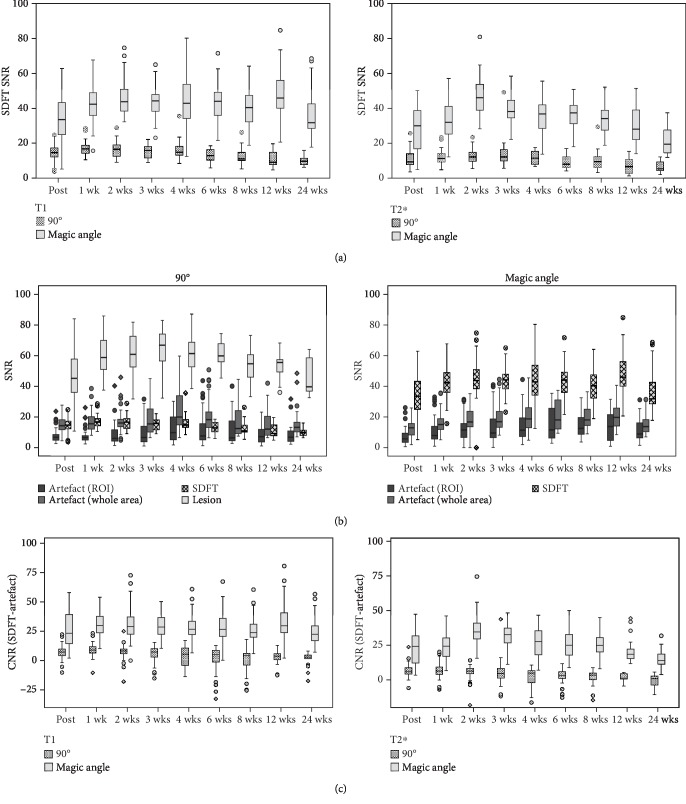
Signal-to-noise ratio (SNR) and contrast-to-noise ratio (CNR) in standard and magic angle images. (a) Boxplots illustrating significantly higher SDFT SNR in magic angle images (*θ* = 55°) compared to standard images (*θ* = 90°) in T1- and T2∗-weighted sequences and at all time points (*P* < 0.001). (b) Boxplots displaying the SNR of the artefact ROI, artefact of the whole area, SDFT, and lesion (only in standard images) in T1-weighted sequences over time. The SNR of hypointense artefacts and SDFT in standard images are similar, making it challenging to distinguish hypointense artefacts from healthy tendon tissue. In contrast, in the magic angle images, SDFT SNR was significantly higher than artefact SNR (*P* < 0.001). Lesion SNR in standard images was significantly higher than artefact SNR as well (*P* < 0.001). (c) Boxplots illustrating the significantly higher CNR between SDFT and artefact in magic angle images compared to standard images in both T1- and T2∗-weighted sequences and at all time points (*P* < 0.001). Circles and rhombs display outlier values; wk: week.

The magic angle effect was observed in all examined SDFT in low-field MRI. We obtained hyperintense signals in the healthy tendon tissue and significantly higher SDFT SNR in magic angle images compared to standard images in both T1- and T2∗-weighted sequences and at all time points (*P* < 0.001) (Figures 3 and 4(a)).

### 3.2. Artefact Discrimination

Evaluation of the blind review showed that category 1 artefacts (images: *n* = 1115) were all part of the image series obtained from limbs injected with SPIO-labelled MSC, which demonstrates a correct identification of SPIO artefacts (Figure 2). However, among category 2 artefacts (images: *n* = 140), there were also images obtained from the control limbs (*n* = 25) or before MSC injection (*n* = 10), thus demonstrating that these images did not reliably display artefacts caused by SPIO-labelled MSC (Figure 2).

**Figure 5:**
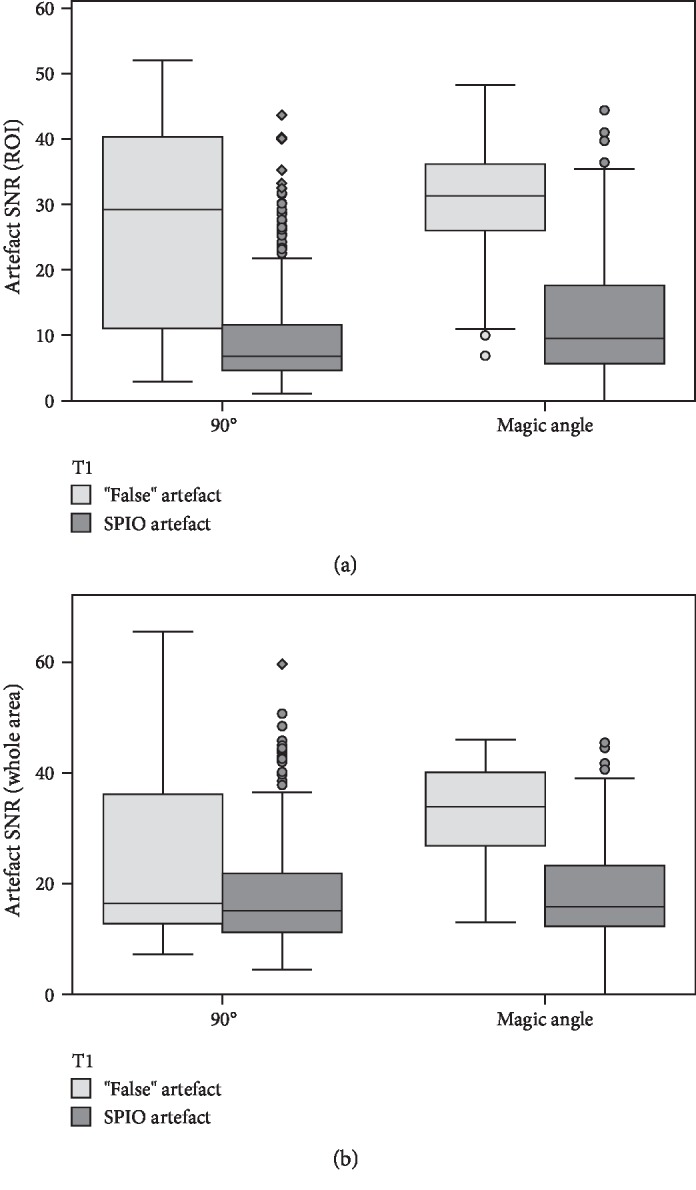
Differences in signal-to-noise ratio (SNR) between SPIO or “false” artefacts. Boxplots exhibiting the comparison of artefact SNR in images categorized as 1 (“hypointense area most likely related to SPIO”=SPIO artefact) and 2 (“questionable hypointense area”=“false” artefact) in T1-weighted sequences obtained at *θ* = 90° or magic angle (*θ* = 55°). It shows that the artefact SNR of the ROI (a) and area (b) in the potentially false-positive category 2 images was not as low as in the category 1 images (*P* < 0.001). However, a threshold SNR value for the discrimination of true SPIO artefacts and other hypointense artefacts was not definable. Circles and rhombs display outlier values; different assessment time points are summarized.

In contrast, artefacts appeared hypointense and with low SNR in both magic angle and standard category 1 images, with no significant differences observed at most time points (Figures 3 and 4(b)). Interestingly, SPIO SNR in the false-positive category 2 images was not as low as in images of category 1 (*P* < 0.001), the difference being most evident in T1-weighted magic angle images (Figure 5). However, a threshold SNR value delimiting SPIO-induced artefacts could not be defined.

All category 2 images were excluded from the following analyses. In standard images, when the SPIO artefact was surrounded by a hyperintense tendon lesion, its borders were clearly definable, but when it was localised within healthy tendon tissue, a clear distinction was only possible in magic angle images. This observation was reflected by the SNR values obtained from the artefact, the lesion, and the SDFT (Figure 4(b)). Lesion SNR measured in standard images was significantly higher than artefact SNR in both T1- and T2∗-weighted sequences and at all time points (*P* < 0.001). On the contrary, SDFT SNR in standard images was in a similar range as artefact SNR, especially considering the values obtained from the whole artefact area. However, in the magic angle images, SDFT SNR was significantly higher than artefact SNR in both T1- and T2∗-weighted sequences and at all time points (*P* < 0.001). This finding corresponds to the comparison of the CNR based on SI SDFT and SI artefact between magic angle and standard images (Figure 4(c)). It was significantly higher in magic angle images in both T1- and T2∗-weighted sequences and at all time points (*P* < 0.001). Nevertheless, despite these differences, artefact volume did not differ significantly between magic angle and 90° images (data not shown).

### 3.3. Follow-Up

Category 1 artefacts could be distinguished at the MSC injection site over the whole follow-up period until week 24. Artefact SNR constantly remained low, and CNR correspondingly remained high and was not influenced by a decreasing signal during tendon healing over time in T1-weighted images. In contrast, T2∗-weighted images showed a decreasing trend in SDFT SNR, which was also evident in magic angle images and reflected by CNR in these images. Nevertheless, the differences in CNR between standard and magic angle images still remained significant until week 24 (*P* < 0.001) (Figure 4).

**Figure 6:**
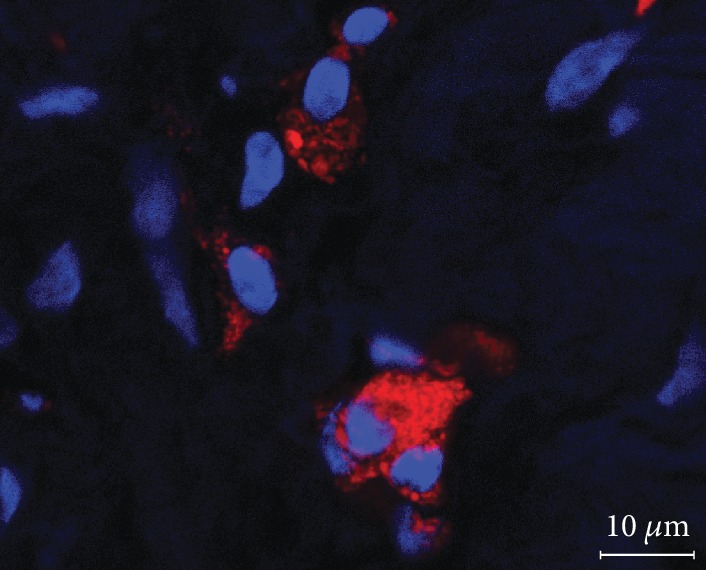
Fluorescence microscopy. Representative image of a tendon lesion 24 weeks after injection of SPIO-labelled MSC with red rhodamine B fluorescence of iron oxide particles and blue DAPI staining of nuclei.

Evaluation of the histological sections revealed that SPIO-labelled cells were still present at the lesion site at week 24, suggesting that the artefacts observed in MRI corresponded to labelled MSC (Figure 6).

## 4. Discussion

The first important finding of this study is that magic angle images can be obtained from the equine SDFT in standing low-field MRI resulting in images with higher SI in the SDFT. Therefore, it can be used for preclinical and clinical research in horses. As demonstrated here, this offers considerable advantages for cell tracking studies. Moreover, magic angle images could be used for more detailed diagnostics in tendon disease [5, 6]. However, while the current data demonstrate the feasibility of this technique, it should be acknowledged that the achievable angle between the SDFT and the main magnetic field is limited by the rigid poles of the used low-field MRI scanner and the animal size. Therefore, the standardisation of image acquisition is challenging and the use of a wedge was not possible. Furthermore, positioning of the animal should be performed with caution and there could be a high risk for injuries for both horse and person. Success and safety may depend on training and temper of the respective horse.

The second important finding of this study is that the magic angle technique improves the accuracy and reliability of SPIO-artefact discrimination in the SDFT. So far, the use of the magic angle effect for cell tracking in vivo had only been reported in one in vivo study in rabbits [14], in which comparison with standard images has not been performed.

Although in this study, the artefact volume determined in the standard image series was similar to those analysed in the magic angle image series, it should not be assumed that the cells are located solely within the tendon lesion at all times. They are likely to be distributed within the healthy tendon around the lesion and the tendon sheath after being injected into the lesion [8, 15, 21]. As demonstrated in our study, SPIO artefacts and healthy tendon tissue display an equally hypointense signal in standard images. Only the tendon lesion displays a higher SNR compared to the artefacts, thus they can be distinguished clearly. However, a reliable identification of the artefact area within the whole tendon is nearly impossible. These issues can be overcome using additional magic angle images to determine the artefact localisation more accurately. In magic angle images, only the SPIO artefacts display a hypointense signal. Therefore, it is possible to distinguish them from the surrounding tendon tissue, which is of particular importance for a detailed tracking of SPIO-labelled MSC.

Interestingly, a subjective blinded review was demonstrated to be surprisingly accurate, as no hypointense areas in control limbs or before MSC injection had been categorised as “most likely related to SPIO.” In contrast, all hypointense areas found in control limbs or before cell injection had been considered as “questionable hypointense areas,” demonstrating a correct identification of potential false-positive artefacts. Unspecific artefacts may arise as a result of high magnetisation at the transition of different tissues. However, due to few outlier SNR values in questionable and SPIO artefacts, it was not possible to define a fixed threshold SNR value delimiting true SPIO artefacts in standard and magic angle MRI images. Possibly, these outlier values in SPIO artefacts were caused by the partial-volume effect in MRI images which leads to the interference of signals and which can therefore increase the SI of especially small artefact areas. Regarding the lack of significant differences between artefact volume, it is a surprising finding and underlines that subjective discrimination of artefacts works well in most cases (as also shown by the subjective artefact categorization). However, the magic angle effect is still very useful in challenging cases.

To confirm that artefacts seen in MRI correspond to SPIO-labelled cells, histology can be considered as a gold standard technique [7, 14, 15]. In the current study, histology showed that SPIO particles were present and localised in vital, spindle-shaped cells up to week 24 (Figure 6), as described in more detail previously [19]. However, while histology remains the most reliable method to validate MRI results, it is obviously not suitable for longitudinal in vivo tracking. Therefore, using MRI to obtain standard as well as magic angle image series, cell tracking was feasible over a long period of 24 weeks. Yet, it should be acknowledged that low-field MRI, despite using the magic angle technique, is not suitable to detect small numbers of labelled cells. Using low-field MRI, 100,000 SPIO-labelled MSC were visible in agar gel but lower concentrations can certainly not be distinguished [8]. The cell concentrations in the tendon decrease over time which is represented by the decreasing artefact volume in MRI and the examination of the histological sections [7, 19]. Besides this, there were different in vivo studies about longitudinal cell tracking of SPIO-labelled MSC in tendons with a detection period of 8 and 9 weeks but without further follow-up [7, 8] and in joints with a detection period of 12 weeks without further follow-up [22].

One limitation of this study was the use of a low-field MRI system, leading to reduced image resolution, relatively wide slice thickness, and risk of motion artefacts. However, due to the 1% risk of mortality, general anaesthesia required for examinations of horses in high-field systems is a major disadvantage [23]. Moreover, the recovery phase may lead to high strains within the SDFT, leading to a possible negative effect on tendon healing. Therefore, particularly for studies involving repeated examinations as well as examining equine patients, the use of standing low-field MRI with a lower risk for the animals is favourable.

## 5. Conclusion

In conclusion, in vivo magic angle imaging has not been described in large animals. The current data show for the first time that the approach is feasible in the horse, which is of high interest for future in vivo studies. At the same time, the results demonstrate the advantages of using the technique in practical implementation for long-term cell tracking.

## Figures and Tables

**Table 1:** Settings of MRI sequences.

Sequence	TR (ms)	TE (ms)	FA (°)	ST (mm)	Gap (mm)	FOV (mm^2^)	Matrix
T1w GRE	52	8	50	5	1	171 × 171	256 × 256
T2∗w GRE	68	13	25	5	1	171 × 171	256 × 256

GRE: gradient echo; TR: time to repeat; TE: time to echo; FA: flip angle; ST: slice thickness; FOV: field of view.

## Data Availability

The data used to support the findings of this study are available from the corresponding author upon request.
